# Proximity in mice induced by an auditory-conditioned stimulus

**DOI:** 10.1038/s41386-026-02492-1

**Published:** 2026-07-15

**Authors:** Wataru Ito, Alexei Morozov

**Affiliations:** 1 Fralin Biomedical Research Institute Center for Neurobiology Research at Virginia Tech Carilion, Roanoke, VA USA; 2 Department of Psychiatry and Behavioral Medicine, Virginia Tech Carilion School of Medicine, Roanoke, VA USA

**Keywords:** Limbic system, Social neuroscience

## Abstract

Social affiliation promotes survival and well-being across species, and proximity to conspecifics is a necessary precondition for affiliative contact. While innate threats reliably increase proximity among conspecifics, whether learned threats have the same effect remains unknown. Here, we report that an auditory conditioned stimulus (CS) induces proximity in mice. In same-sex dyads, fear-conditioned mice increased proximity during CS presentation, independent of freezing levels. This CS-evoked proximity required familiarity between partners and intact basolateral amygdala-to-ventral hippocampus inputs, demonstrated by DREADD-mediated suppression. It also required oxytocin receptor signaling, demonstrated by systemic administration of the antagonist L368,899. These findings suggest that learned and innate threats engage shared neural circuitry for social proximity.

## Introduction

Affiliation encompasses a set of social behaviors — including mating, territorial defense, thermoregulation through huddling, skill acquisition, and collective defense against predators — that collectively support survival and well-being [1–4]. All such behaviors involve proximity among conspecifics as an inevitable precondition that enables social contact. Classic fear-affiliation theory, proposed by Schachter, suggests that perceived threat actively drives social contact, particularly with others facing the same danger [5, 6]. Consistent with this, defensive aggregation is observed across species in response to innate threats [7–15], and laboratory studies show that predator cues induce aggregation in rats via neuropeptide systems, including oxytocin and vasopressin [16–18]. Together, these findings suggest that proximity-seeking under threat reflects an adaptive coupling between threat-processing and social-motivational systems, the disruption of which is particularly relevant to psychiatric disorders.

A key unanswered question is whether this coupling extends to learned threat predictors. Do conditioned threat cues increase proximity between conspecifics, and if so, which circuits and molecules mediate this effect? Here, we demonstrate that an auditory CS triggers proximity in fear-conditioned mice tested as same-sex dyads, independent of freezing. This behavior requires basolateral amygdala-to-ventral hippocampus circuitry and oxytocin receptor signaling.

## Materials and methods

All animal procedures were conducted in accordance with the Virginia Tech IACUC-approved protocol 24-110.

### Animals

129SvEv/C57BL/6N F1 hybrids were produced in-house from breeding trios consisting of one C57BL/6N male and two 129SvEv females, weaned at postnatal day 23–28 (p23–28), and housed as four same-sex littermates per cage [19]. Mice were tested at p75–90. Familiar dyads were assembled 7 days before testing from cagemate littermates and housed in fresh bedding that remained unchanged until testing completion. Unfamiliar dyads were assembled at the time of testing by pairing non-littermate mice that had never been co-housed.

### Fear conditioning

Each mouse was conditioned individually in standard chambers (Med Associates, St. Albans, VT) [20] using four pairings of a conditioned stimulus (CS: 30 s, 8 kHz, 80 dB tone) with an unconditioned stimulus (US: 0.5 mA, 0.5 s footshock co-terminating with the CS), presented at variable intervals (60–180 s). Testing was conducted 1–2 days later in a novel context, with two mice per chamber. Each test session lasted 3 min: 1 min baseline (pre-CS) followed by 2 min of CS presentation. Videos were recorded at 4 frames per second and exported as AVI files with MJPEG compression using Freezeframe software (Actimetrics, Wilmette, IL), then converted to MP4 format using a custom Python script.

### Animal tracking and distance estimation

Using a custom Python script, we manually annotated the snout position of each animal in every video frame and recorded the pixel coordinates. Real-world X–Y coordinates of each snout’s projection onto the chamber floor were computed using a triangulation-based geometric transformation. This transformation utilized the pixel coordinates of the snout and three chamber corners, along with the known real-world coordinates of those corners on the chamber floor plane. Intermouse distances were calculated from the resulting X–Y coordinates.

### Quantification of freezing, body-touch, and snout-to-snout contacts

Freezing was defined as the absence of observable movement of the body and vibrissae (except respiration) for at least 1 s [21]. Body-touch was defined as passive body-to-body contact [22]. However, we treated snout-to-snout contacts as independent events. Trained observers manually recorded the first and last video frames of each behavioral bout using a custom Python script, generating time-series data. Freezing percentage was computed from these data using another Python script. Averaged CS-induced freezing across all groups ranged from 37 to 55% and did not differ significantly between treatment conditions within each experiment (Supplementary Table 1).

### Binary viral injections for DREADD inactivation

To selectively express hM4D(Gi) in specific neuronal projections, we used a binary viral strategy. Pseudotyped AAV5-hSyn-DIO-hM4D(Gi)-mCherry (Addgene 44362) was injected at the soma region of interest, and retrograde AAV-hSyn-Cre (Addgene 105553) was injected at the axon terminal region for retrograde transduction. Both AAV stocks were diluted to 5×10^12 viral particles per mL, and 0.2 μL was injected bilaterally at each site. Injection coordinates (AP/ML/DV mm from bregma and brain surface) were as follows: ventral hippocampus (site 1: −3.4/ ± 3.6 / − 3.3, and site 2: −3.6/ ± 2.95/ − 4.5), basolateral amygdala ( − 1.7/ ± 3.2/ − 4.3), and nucleus accumbens ( + 1.4/ ± 0.8/ − 4.1). Surgical procedures were performed according to established protocols [23]. After completion of behavioral testing, the animals underwent intracardiac perfusion with 4% paraformaldehyde; their brains were postfixed overnight and sectioned into 0.15-mm slices using a Vibratome. The expression of hM4D(Gi)-mCherry in the targeted areas was verified by the presence of the somatic red fluorescence.

### Data analysis

Statistical analyses were performed using GraphPad Prism 5 (GraphPad Software, La Jolla, CA). Because CS-induced affiliations were observed in both male and female dyads, with no sex differences (Supplementary Fig. 1A), we combined male and female data for analysis. We limited some DREADD experiments to female dyads only (Fig. 2C, D). Normality was assessed using the Shapiro–Wilk test. Some datasets exhibited non-normal distributions and were analyzed using the Wilcoxon signed-rank test (paired comparisons) and Mann–Whitney test (unpaired comparisons). All tests were two-sided. Interactions between factors were assessed using two-way repeated-measures ANOVA. Statistical significance was set at *p* < 0.05.

We implemented a custom Python analysis pipeline to assess bimodal distributions and present the probability density. To test for bimodality, we employed Gaussian Mixture Modeling (GMM) using the scikit-learn library. We fit each dataset into two competing models: a unimodal (1-component) model and a bimodal (2-component) model. Model performance was evaluated using the Akaike Information Criterion (AIC) and the Bayesian Information Criterion (BIC) by transforming the raw AIC and BIC scores into Akaike weights [24].

## Results

### Auditory conditioned stimulus facilitates proximity between familiar mice

**Fig. 1:**
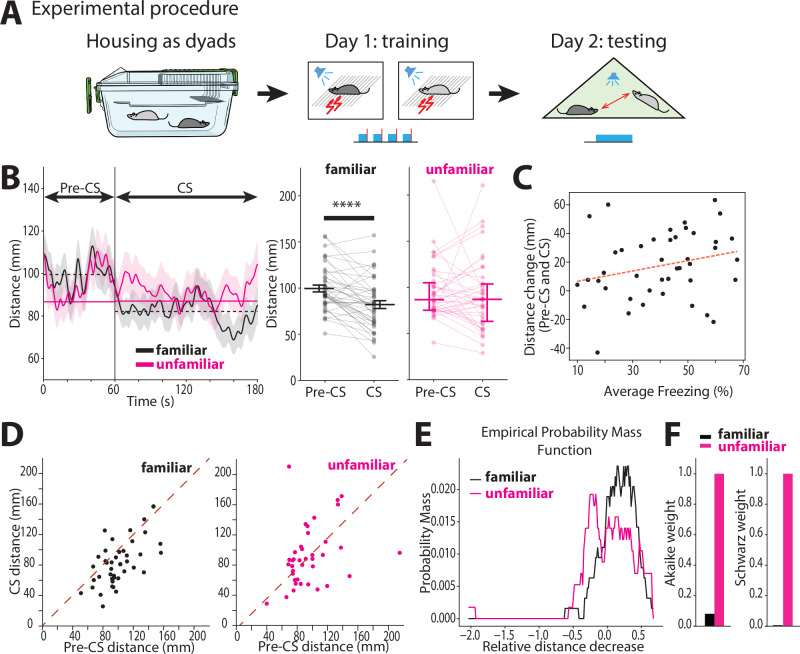
CS-induced social proximity in dyads requires familiarity and is independent of freezing level. **A** Experimental procedure. **B** Left: dyad distance dynamics throughout pre-CS and CS periods. The solid lines represent mean distances (smoothed by a Gaussian filter, the kernel SD = 5). The shaded areas depict SEMs. The solid and broken horizontal lines represent the medians and means, respectively, for the average dyad distances as analyzed in the paired analyses on the right. Right: average distance during pre-CS and CS periods. **Black:** familiar dyads, *n* = 43 (male:20, female:23), paired t-test: ^****^p < 0.0001. **Pink:** unfamiliar dyads, *n* = 39 (male:19, female:20), Wilcoxon Signed Rank test: NS. **C** No correlation between the degree of affiliation (dyadic distance change) and the averaged freezing levels for dyads (Pearson’s coefficient *r* = 0.251, *p* = 0.104). **D** Scatter plot analysis of average distances during the pre-CS vs. CS period. **E** Distributions of the relative CS-induced decreases in distances are plotted as the Empirical Probability Mass Function (ePMF), with the smoothing window defined as 10% of the data range. **F** Quantification of population bimodality. Bars represent the Akaike weights (wAIC) and the Schwarz weights (wBIC) for the 2-component (bimodal) Gaussian Mixture Model fits. Weights near 1 indicate decisive relative support for a bimodal model compared to a unimodal (1-component) null model.

To investigate whether a conditioned stimulus (CS) drives proximity, we housed male and female mice as same-sex dyads and subjected them individually to auditory fear conditioning. Dyads were then tested together by exposing them to the CS in a novel context (Fig. 1A). Following a 1-min baseline (pre-CS) period, the CS was presented for 2 min.

During the test, mice did not exhibit affiliative behaviors toward conspecifics, such as grooming, huddling, or mounting. Most physical contacts were passive and non-directed [22] — brief, incidental body-to-body encounters that did not conform to the structured social interaction categories typically observed in the home cage [25]. We therefore termed these contacts ‘touching’.

CS moderately increased body-touching in both % time and bout duration, while naturally decreasing bout counts/min. **(**Supplementary Fig. 1A**)**. Animals also exhibited rare conspecific-directed behaviors, snout-to-snout contacts [22]. For these snout contacts, the medians of the % time or their duration showed tendencies toward a decrease by CS. Consistently, CS significantly decreased the number of contacts/min **(**Supplementary Fig. 1B**)**. Notably, we observed that animals decreased the distance aiming toward each other’s snouts, even without contacts.

Therefore, we quantified proximity by measuring snout-to-snout distance. This measure is particularly relevant given that rodent head movements direct gaze [26] and whisker aiming reflects spatial attention [27]. During the CS presentation, snout-to-snout distance decreased significantly compared to the pre-CS period (*p* < 0.0001, Fig. 1B), indicating increased proximity between partners. This effect was independent of sex (Supplementary Fig. 2A). To determine whether freezing behavior could account for the observed decrease in distance, we examined the relationship between freezing levels and changes in distance. These variables showed no correlation (Fig. 1C), suggesting that independent mechanisms mediate CS-evoked proximity and freezing. Nevertheless, we summarized freezing levels across all experimental groups in Supplementary Table 1 and found no significant differences between groups within each experiment.

We next tested whether the decrease in distance was a time-dependent process unrelated to CS. A control group of fear-conditioned mice was tested in the absence of CS presentation. These dyads showed no decrease in snout-to-snout distance between the first and last two minutes of the session (Supplementary Fig. 2B), demonstrating that CS presentation is necessary for the proximity.

Finally, we examined whether familiarity between partners influences CS-induced proximity. When dyads were formed from unfamiliar mice that had not been co-housed, CS presentation failed to decrease snout-to-snout distance (Fig. 1B, Supplementary Fig. 2A). This result indicates that familiarity is required for CS-induced proximity, paralleling findings of a better coordination of innate threat responses among familiar conspecifics in different organisms [28–30].

Notably, unlike in familiar mice, the CS effect, assessed as the relative CS-induced decrease in distance (pre-CS distance - CS distance)/pre-CS distance, exhibited a non-normal distribution (Shapiro–Wilk normality test; unfamiliar: W = 0.79, p < 0.0001; familiar: W = 0.98, *p* = 0.78). We predicted that unfamiliar dyads comprise subgroups with distinct responses to CS. The scatter plot of CS vs. pre-CS distances in familiar mice revealed a single cluster centered below the unity line, indicating a uniform CS-induced approach response **(**Fig. 1D**, left)**. In contrast, the unfamiliar group showed a dispersed distribution **(**Fig. 1D**, right)**, suggesting heterogeneity in the CS effect across dyads.

Further, we examined the distribution of the relative CS-induced decrease in distance using an empirical probability mass function (ePMF). The familiar group displayed a single density peak, whereas the unfamiliar group showed two prominent peaks **(**Fig. 1E**)**. To formally assess bimodality, we fit both unimodal and bimodal Gaussian Mixture Models (GMM) to each distribution and compared them using Akaike weights (*wAIC*) and Schwarz weights (*wBIC*) for the bimodal model, which reflect the relative probability that the bimodal fit is the most parsimonious model [24]. In the familiar group, the bimodal model received little support (*wAIC* = 0.081, *wBIC* = 0.006). In the unfamiliar group, however, there was decisive evidence for bimodality (*wAIC* > 0.99, *wBIC* > 0.99) **(**Fig. 1F**)**. Together, these results indicate that the unfamiliar group comprises two distinct sub-populations of dyads with divergent responses to the CS.

### Identification of neuronal circuits required for CS-induced proximity

The amygdala assigns negative valence to auditory stimuli during fear conditioning and responds to the conditioned stimulus (CS) during memory retrieval [31, 32]. The amygdala also exerts opposing or dual effects on social approach and avoidance in a context-dependent manner. For example, basolateral amygdala (BLA) promotes social behavior via 5-HT1A receptor signaling [33] and secretin-expressing neurons [34], while driving social avoidance through projections to the vHPC [35] and NAc [36]. Among BLA downstream targets, the vHPC responds to social cues with increased firing in more than 25% of its neurons [37] and drives social approach via outputs to the lateral septum [38] and NAc shell [39]. The NAc is implicated in a broad range of affiliative behaviors [40–43]; notably, oxytocin signaling within the NAc promotes approach toward a preferred conspecific in mandarin voles [44], and the NAc shell hypocretin system regulates social approach in California mice [45]. Importantly, NAc neurons are recruited during fear expression [46–48] and contribute to transitions between appetitive and escape behaviors in stressful environments [49], suggesting a potential role in CS-induced proximity. Together, these observations led us to test the hypothesis that CS-activated BLA neurons modulate proximity through specific downstream targets.

We employed chemogenetic inhibition of BLA projections to each target. Before conducting pathway-specific manipulations, we verified that clozapine-N-oxide (CNO) itself does not affect CS-induced proximity. Control dyads without expressing DREADD effector were injected with either CNO or vehicle (saline) and showed equivalent decreases in snout-to-snout distance in response to the CS (Supplementary Fig. 3), ruling out non-specific effects of CNO.

### BLA→vHPC pathway

**Fig. 2:**
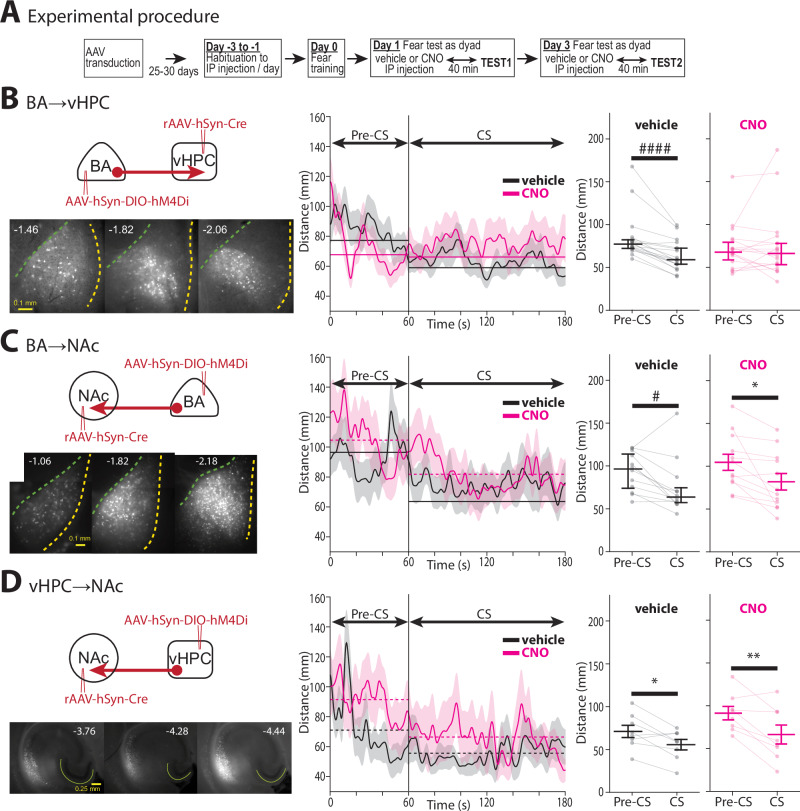
Amygdala-hippocampal projection neurons are required for CS-induced proximity. **A** Experiment timeline. We tested Amygdala-hippocampal (BA➜vHPC) **B**, Amygdala-Nucleus Accumbens (BA➜NAc) **C** and Hippocampal-Nucleus Accumbens (vHPC➜NAc) **D** projections in the same way. (Left Upper) Binary strategy to express hM4Di-mCherry in each projection. Red-filled circle and arrow represent hM4Di-mCherry expressing cell body and axon. (Left Lower) mCherry fluorescence in the cell bodies of targeted neurons, **B**,**C** BA coronal and **D** vHPC horizontal slices. The yellow and green dashed lines indicate the lateral and medial boundaries of BLA **B, C**. The yellow lines mark the dentate gyrus **D**. (Center) Distance dynamics throughout the pre-CS and CS periods. (Right) Average distance during pre-CS and CS periods. Details are the same as in Fig.1B. (B, BA➜vHPC) n = 18 dyads (male:10, female:8), vehicle: ^####^*p* < 0.0001 and CNO: NS (both Wilcoxon signed rank test). (C, BA➜NAc) *n* = 12 female dyads, vehicle: ^#^*p* < 0.05 (Wilcoxon) and CNO: ^*^p < 0.05 (Paired t-test^)^. (**D**, vHPC➜NAc) *n* = 8 female dyads, vehicle: ^*^p < 0.05 and CNO: ^**^p < 0.01 (both Paired t-test).

We first tested the BLA➜vHPC pathway by expressing hM4Di in BLA neurons projecting to vHPC using a binary viral strategy (Fig. 2B). Dyads received intraperitoneal injections of either vehicle or CNO 40 min before CS testing, with each dyad tested twice under both conditions, separated by a two-day interval (Fig. 2A). A two-way repeated-measures ANOVA revealed a significant interaction between the CS and CNO effects on distance (F(1,34) = 9.59, *p* = 0.0039). Under vehicle conditions, dyads exhibited a robust CS-induced reduction in distance (*p* = 0.003). In contrast, CNO treatment decreased baseline pre-CS distance (*p* = 0.010, Wilcoxon signed-rank test) and abolished the CS-induced distance reduction (Fig. 2B). Thus, the BLA➜vHPC pathway contributes to maintaining baseline distance and CS-induced proximity.

### BLA→NAc pathway

We next tested the BLA➜NAc pathway using the same approach in female dyads (Fig. 2C). A two-way repeated-measures ANOVA detected no CS×CNO interaction. Dyads exhibited significant CS-induced distance reduction regardless of treatment (vehicle: p = 0.027; CNO: p = 0.011), and CNO did not affect baseline pre-CS distance. These results indicate that the BLA➜NAc pathway is not required for CS-induced proximity.

### vHPC→NAc pathway

Given that the vHPC➜NAc pathway regulates approach/avoidance behaviors toward conspecifics [39, 50] and we confirmed that the BLA➜vHPC Pathway influences intermouse distance (Fig. 2B), we tested the role of vHPC neurons projecting to NAc using the same chemogenetic approach in female dyads (Fig. 2D). A two-way repeated-measures ANOVA detected no CS×CNO interaction. Dyads exhibited significant CS-induced distance reduction under both vehicle (*p* = 0.039) and CNO (*p* = 0.023) conditions. However, CNO treatment increased baseline pre-CS distance relative to vehicle (*p* = 0.022, Paired t-test; Fig. 2D). Thus, the vHPC➜NAc pathway contributes to baseline distance maintenance but does not to CS-induced proximity.

### Oxytocin signaling is required for CS-induced proximity

Oxytocin is known to enhance the salience of social stimuli [51] and supports social behaviors [52], but exerts two opposing effects — promoting approach or avoidance — depending on the brain site of action and recruited signaling pathway [43, 53]. Here, we addressed whether oxytocin contributes to CS-induced proximity.

**Fig. 3:**
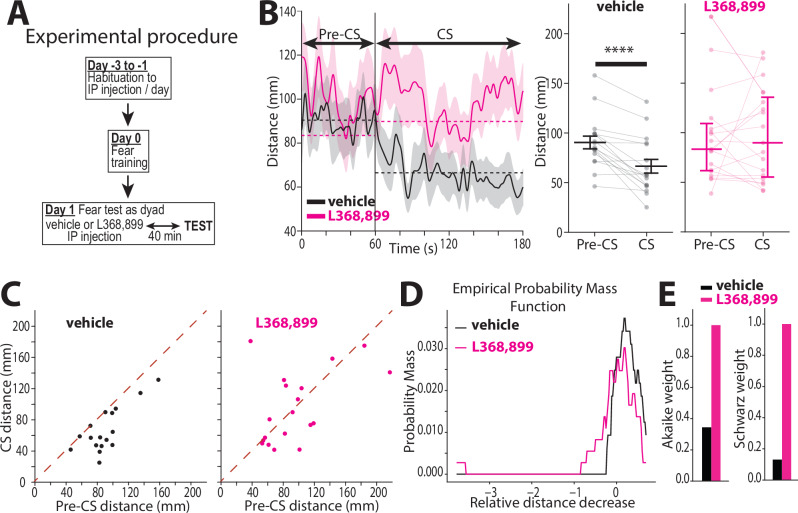
Oxytocin receptor antagonist L368,899 prevents CS-induced proximity. **A** Experimental timeline. **B** Distance dynamics throughout the pre-CS and CS periods. (Right) Average distance during pre-CS and CS periods. Details are the same as in Fig.1B. Vehicle: *n* = 17 dyads (male:9, female:8), ^****^p < 0.0001 (Paired t-test). L368,899: *n* = 19 dyads (male:9, female:10), no marking as NS (Wilcoxon signed rank test). **C** Scatter plots of average distances during CS vs. pre-CS. **D** Distributions of the relative CS-induced decreases in distances are plotted as the Empirical Probability Mass Function (ePMF). The details are the same as in Fig. 1E. **E** Quantification of population bimodality. Bars represent the Akaike weights (wAIC) and the Schwarz weights (wBIC) for the 2-component (bimodal) Gaussian Mixture Model fits. The details are the same as in Fig. 1F.

We administered the oxytocin receptor antagonist L368,899 (3 mg/kg, intraperitoneal) or saline 40 min before CS testing, at the dose shown to modulate social behaviors in mice [53–55]. Each dyad was tested once under either condition (Fig. 3A).

L368,899 did not affect baseline intermouse distance during the pre-CS period (*p* > 0.05, Mann-Whitney U test). A mixed-model ANOVA revealed a CS×treatment interaction approaching significance (F(1,34) = 3.81, *p* = 0.06); CS presentation significantly decreased intermouse distance in saline-treated dyads (*p* = 0.0001), but not in antagonist-treated dyads (*p* > 0.05). (Fig. 3B), demonstrating that oxytocin receptor signaling is necessary for CS-induced proximity.

Notably, while the saline group showed a normal distribution of CS-induced relative distance changes, the L-368,899-treated dyads exhibited a non-normal distribution (Shapiro–Wilk: saline: W = 0.95, p = 0.38; L-368,899: W = 0.55, p < 0.0001), mirroring the pattern observed in unfamiliar dyads. Further, scatter plots of CS versus pre-CS distances and the ePMF analysis revealed that the L-368,899 group was more broadly dispersed than the saline group (Fig. 3C) and exhibited a more skewed density peak (Fig. 3D), resembling the pattern seen in unfamiliar mice (Fig. 1D, E). Finally, Gaussian mixture model fitting with AIC- and BIC-based model selection found little support for bimodality in the saline group (*wAIC* = 0.35, *wBIC* = 0.13) but decisive evidence for it in the L-368,899 group (*wAIC* > 0.99, *wBIC* > 0.99) **(**Fig. 3E**)**, indicating that oxytocin receptor blockade unmasks two distinct subpopulations of dyads with divergent responses to the CS.

To determine whether L-368,899 has a general effect on interanimal distance under our testing conditions—independent of CS-US association or the aversive experience of US exposure—we trained a separate cohort of mice by exposing them to the CS while omitting the US. These dyads underwent the fear-conditioning test twice, on days 1 and 3 after training, receiving L-368,899 or vehicle in a pseudo-random order (Supplementary Fig. 4A). Animals did not freeze, consistent with the lack of US during training. Distances did not differ significantly between L-368,899- and vehicle-injected dyads during either the pre-CS or CS period, nor between the pre-CS and CS periods (Supplementary Fig. 4B). This control indicates that, in the absence of CS-US associative training, neither L-368,899 nor the CS significantly affects interanimal distance.

## Discussion

This study demonstrates that the auditory conditioned fear response in dyads includes not only freezing but also a decrease in physical distance between familiar partners. This CS-induced proximity occurs independently of freezing levels and requires the BLA➜vHPC pathway and oxytocin receptor signaling.

Defensive aggregation is a widespread phenomenon in which threatened animals reduce individual risk by decreasing interindividual distance [56]. While defensive aggregation has been extensively documented in response to innate threat stimuli, particularly predator-related cues [7–15], our findings demonstrate for the first time that a learned threat signal—an auditory CS—can also elicit proximity-seeking in same-sex dyads of laboratory mice. This finding reveals that the conditioned fear response encompasses both non-social defensive behaviors, such as freezing, and social approach behaviors. However, these two types of defensive responses occur independently, as evidenced by the lack of correlation between them and the absence of freezing alterations across all circuit manipulations (Supplementary Table 1). This independence suggests that CS-activated freezing pathways, which include connections from the lateral to the central amygdala [57, 58], operate independently of CS-activated social proximity circuits.

The requirement for familiarity in CS-induced proximity implicates social recognition systems, including the hippocampus, which mediates social memory [50, 59]. Chemogenetic inhibition of the BLA➜vHPC pathway abolished CS-induced proximity without altering freezing, consistent with a model in which the amygdala detects the threatening CS and transmits this information to the hippocampus to drive approaching behavior. However, this circuit silencing also increased baseline proximity during the pre-CS period, suggesting that tonic BLA➜vHPC activity suppresses proximity in the absence of threat. This interpretation aligns with findings that optogenetic inhibition of BLA➜vHPC projections increases social approach [35]. These observations argue against a simple model in which “CS-activated amygdala drives proximity”. Instead, CS presentation may suppress a subset of BLA neurons projecting to vHPC, thereby disinhibiting affiliative behavior. This interpretation is supported by evidence that CS upregulates some BLA neurons while downregulating others [31], and that BLA sends both inhibitory and excitatory projections to vHPC with potentially distinct CS responses [60]. Future studies monitoring BLA➜vHPC projection dynamics during CS presentation will be necessary to test these possibilities.

Inhibition of BLA➜NAc projections did not affect either CS-induced proximity or baseline intermouse distance, which was unexpected given evidence that the NAc regulates social approach [43, 44, 61] and BLA inputs to NAc regulate sociability [36]. In contrast, inhibiting vHPC➜NAc projections increased baseline intermouse distance, consistent with reports that vHPC➜NAc pathway activity correlates with social interaction in mice [62] and that its inhibition decreases social interaction in rats [39]. However, this manipulation did not prevent CS-induced proximity, suggesting that CS recruits a parallel pathway that bypasses or overrides this circuit. The vHPC projections to the lateral septum are promising candidates, given their roles in regulating approach-avoidance decisions toward novel conspecifics [38] and in motivational conflict [63], and the finding that rats showing greater predator odor-induced aggregation exhibit elevated c-Fos expression in the lateral septum [17].

We tested the role of oxytocin signaling using systemic administration of the high-affinity oxytocin receptor antagonist L-368,899, which crosses the blood-brain barrier [53] and modulates social behaviors in mice [53–55]. At the group level comparison, the antagonist abolished CS-induced proximity, consistent with evidence across species that oxytocin and its homologs promote inter-organism distance reduction under threat, including flocking in birds [64], shoaling in fish [65] and predator-odor-induced aggregation in rats [18].

However, analyses of sample distributions revealed that oxytocin receptor blockade did not uniformly suppress proximity-seeking; instead, it unmasked divergent responses across dyads, best described by a bimodal distribution reflecting two distinct subpopulations. Similar biomodal distributions were observed in the unfamiliar dyad group. We speculate that this heterogeneity is attributable to individual differences in social recognition and approach circuitry, including variation in oxytocin receptor expression and activity.

Because L-368,899 was administered systemically, the circuit locus of oxytocin’s effect on CS-induced proximity remains unidentified. Studies in prairie voles and California mice have identified the NAc, anterior cingulate cortex, and BLA as sites where oxytocin regulates social approach [43, 66]. Whether oxytocin acts within the BLA-to-vHPC circuit identified here, or at parallel sites, remains to be determined through circuit-specific manipulation of oxytocin receptor signaling.

## Supplementary information

Supplementary materials

## Data Availability

All primary data, including video files, are available from the authors upon reasonable request. Analysis code and example datasets will be provided as part of a replication package upon publication.

## Code availability

All primary data, including video files, are available from the authors upon reasonable request. Analysis code and example datasets are available at https://github.com/wataruito/Codes_in_Threat-induced_proximity_Ito_et_al.
