# Supplementary material for: Proximity in mice induced by an auditory-conditioned stimulus
Source: Neuropsychopharmacology. 2026 Jul 15;51(10):1836–43. doi: 10.1038/s41386-026-02492-1 (PMC13486637; doi:10.1038/s41386-026-02492-1)
Supplement: Supplementary file 1 [file 41386_2026_2492_MOESM1_ESM.pdf]

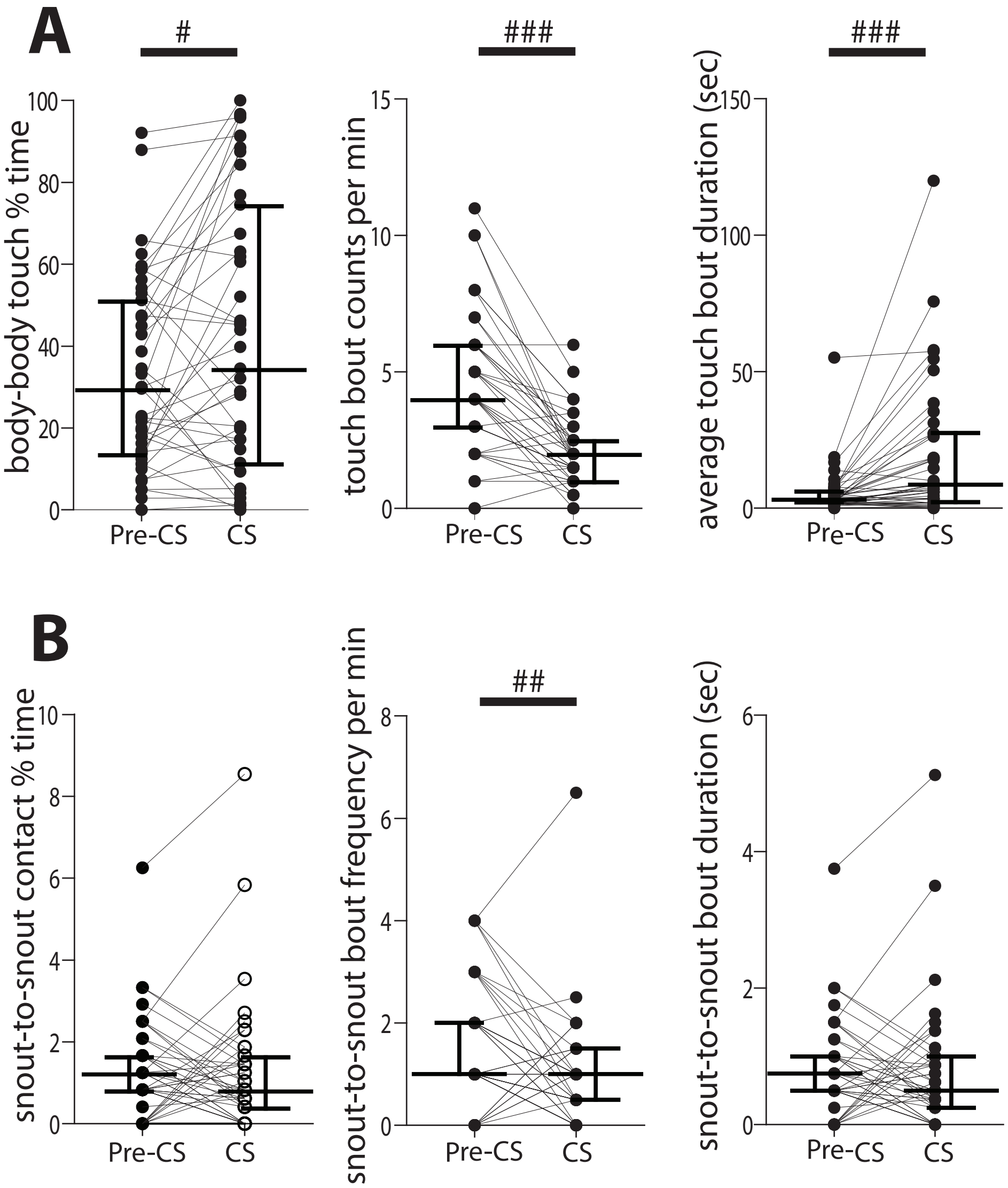

Supplementary Figure 1. Contacts during pre-CS and CS phases of fear testing. (A) Passive body-body touch % time (left), touch bout counts/min (middle) and averaged touch bout duration (right). (B) Same measures for snout-to-snout contacts. Wilcoxon Signed Rank test: #p<0.05, ##p<0.01, ###p<0.001, n=43 dyads). Medians and interquartile ranges are shown.

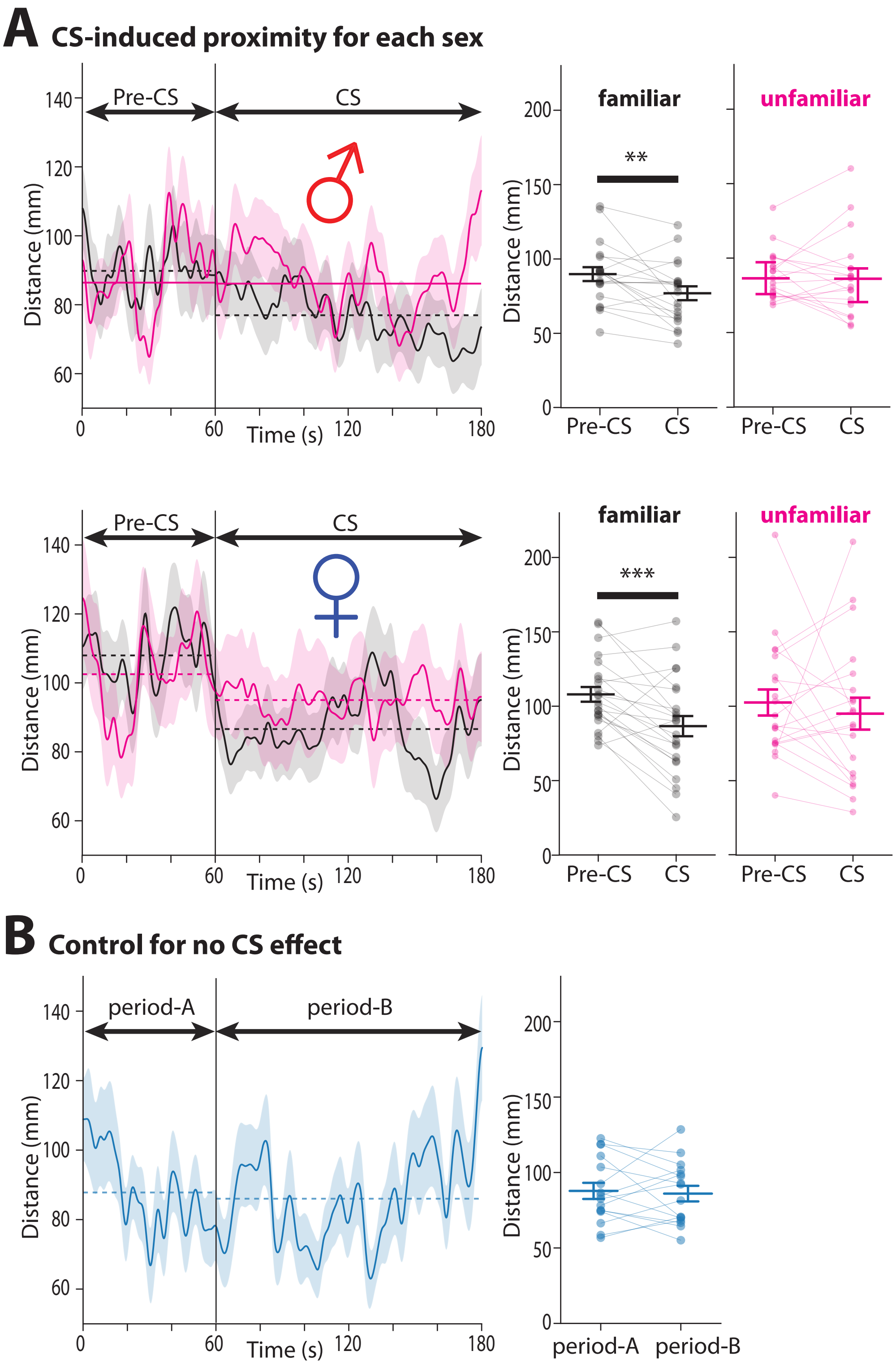

Supplementary Figure 2. Data split by sex and controls for the experiments in Figure 1. (A) Both sexes exhibit similar CS-induced proximity. Upper: male same-sex dyads (familiar/unfamiliar = 20/19 dyads). Lower: female same-sex dyads (familiar/unfamiliar = 23/20 dyads). Details are the same as in Figure 1B. Unfamiliar male dyads: Wilcoxon Signed Rank test: NS. The remaining all groups: Paired t-test: **p<0.01, ***p<0.001 and no marking as NS. (B) CS is required for the proximity. Separate cohort of familiar dyads tested without CS (n=16 dyads, male:9, female:7). Paired t-test: NS.

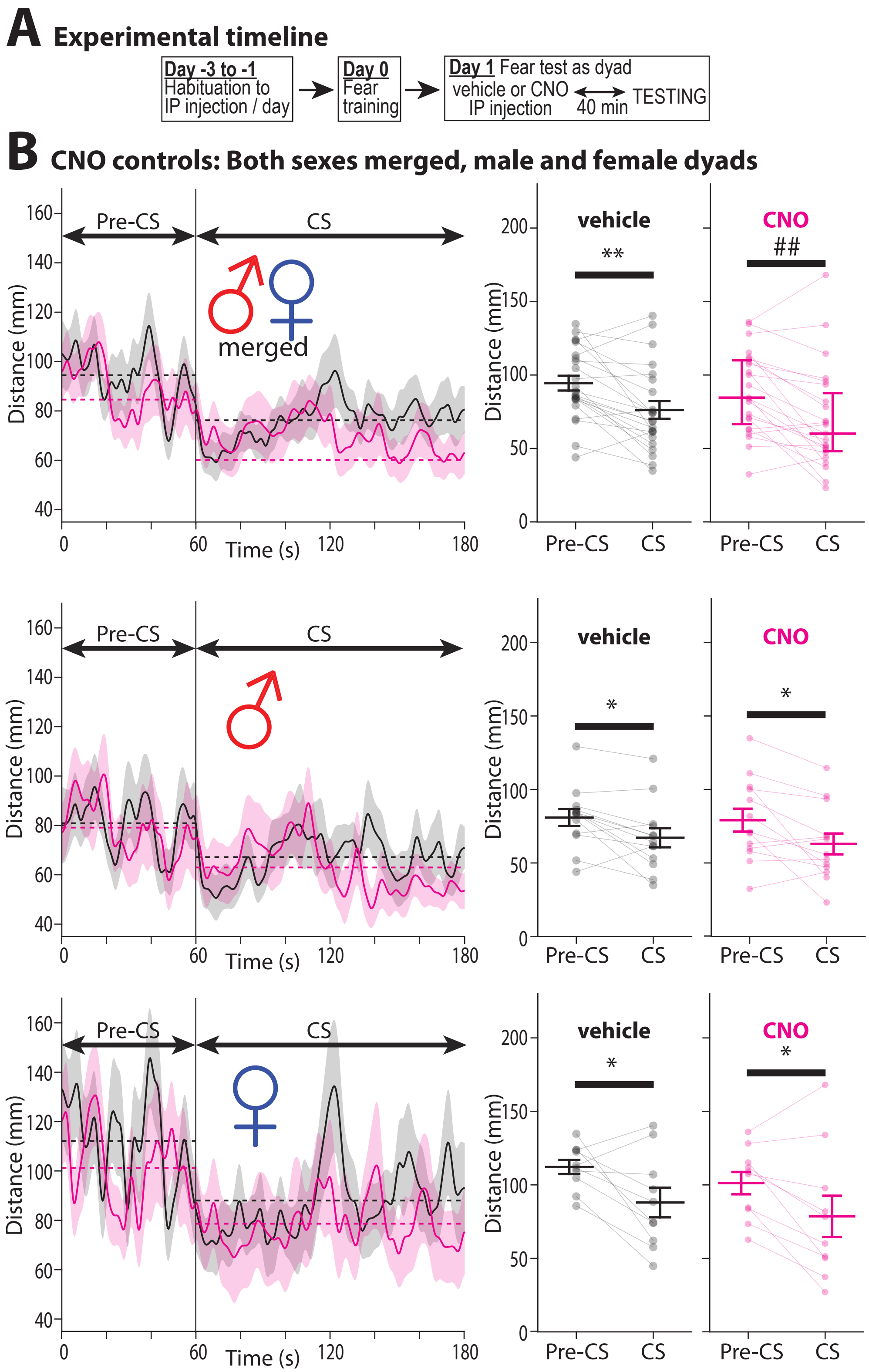

Supplementary Figure 3. CNO alone does not affect CS-induced proximity. (A) Experimental timeline. (B) Distance dynamics across the pre-CS and CS periods in merged male and female dyads (n=23). The solid lines represent mean distances smoothed by a Gaussian filter (the kernel SD = 5). The shaded areas depict SEMs. (C) Average distance during pre-CS and CS periods. Data split by sex is shown in D, E (females, n=10) and F, G (males, n=13). Paired t-test: *p<0.05, **p<0.01, Wilcoxon signed rank test: ##p<0.01.

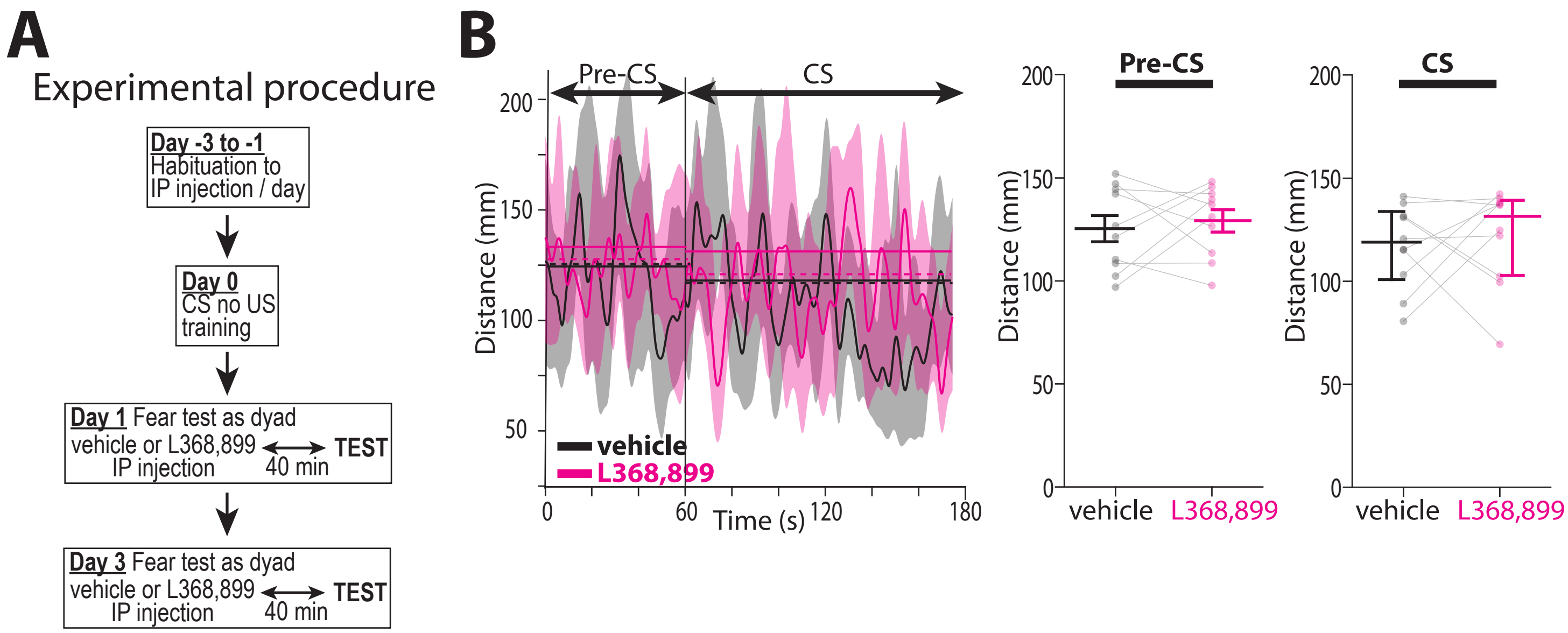

Supplementary Figure 4. L-368,899 and the CS do not affect proximity in dyads trained without US. (A) Experimental timeline. (B) Left: Distance dynamics throughout the pre-CS and CS periods. Solid lines represent mean distances (smoothed with a Gaussian filter; kernel SD = 5), and shaded areas depict SEMs. Solid and broken horizontal lines represent the medians and means, respectively, of the average dyad distances analyzed in the paired comparisons on the right. Right: Average distance during the pre-CS and CS periods in dyads injected with vehicle or L-368,899 (n = 10 male dyads). Means ± SEM are shown for the pre-CS comparisons and medians ± interquartile ranges for the CS comparisons. Paired t-tests and Wilcoxon signed-rank tests detected no significant effect of L-368,899 or the CS on distance.

Supplementary Table 1. Summary of freezing levels for each experiment.

GROUP	TREATMENT	% FREEZING	COMPARISON
familiar	none	40±16	ns
unfamiliar		41±18	
BLA->vHPC	vehicle	42±20	ns
	CNO	39±21	
BLA->NAc	vehicle	40±14	ns
	CNO	43±17	
vHPC->NAc	vehicle	52±17	ns
	CNO	37±18	
for vehicle	vehicle	53±18	ns
for L368,899	L368,899	55±20	
CNO control	vehicle	53±18	ns
	CNO	55±20	
